# Fossil Remains

**Published:** 1845-09

**Authors:** 


*Fossil Remains.*—In the last No. of the American Journal of Science and Arts, are several paragraphs on recent discoveries of fossil animals, that fill the naturalist with perfect astonishment. For example, at Algoa Bay, near the Cape of Good Hope, several skulls, together with bones of the extremities, have been found, which belonged to several species of reptiles. Some of them appear to hold a place between the tortoise and lizard. The fact seems to be settled, that a reptile once existed which had only two teeth.

A large skeleton of the Zeuglodon was brought to light some months since in Alabama, by Dr. Albert C. Kock. One of those mighty skeletons, about seventy-feet in length, is said to be in Albany at the present moment. But Dr. Kock's discovery puts all former ones into the shade, his skeleton being *one hundred and four feet long!* The solid portions of the vertebræ are from fourteen to eighteen inches in length, from eight to twelve in diameter, and weigh, upon an average, seventy-five pounds. There are forty incisor teeth, four canine and eignt molars. It is decided that the monster was a carnivorous one. The eyes were large and prominently situated, says the Journal, on the forehead, giving the animal the power of keeping a constant and vigorous watch for its prey.

Lastly, in the category of anomalous things, by way of remembrances of an old world, of which these colossal bones are the mere fragments, some parts of the skeleton of the extinct gigantic New Zealand bird, called moa, were carried to England in February last, and offered for sale to the College of Physicians. The owner asked for the lot, the round sum of two hundred pounds sterling, which the learned gentlemen of the institution were not inclined to give, and they were therefore intended for a Paris market.

From the deep interest everywhere manifested in that class of researches which bring up and bring out of the earth the unquestioned evidence of the fact, that the surface of the globe has in ages of undefined remoteness, been in the entire occupancy of monsters of the hugest and strangest proportions, but which were in exact adaptation to the physical condition of things in the ages in which they lived, we may hope for revelations of a still more exciting character. As men multiply, and, prompted either by the passion of curiosity, ambition or avarice to penetrate the earth's crust, new developments and strange discoveries are in all probability constantly to be made. In all past periods much has been lost through ignorance; yet gains are to be hoped for in future, since comparative anatomy comes with all the laws explanatory of animal architecture, to illustrate and establish the great truths discoverable in the vestiges of creation.—*Boston Med. and Surg. Journal.*

